# The Interplay of Electronic Configuration and Anion Ordering on the Magnetic Behavior of Hydroxyfluoride Diaspores

**DOI:** 10.1021/acs.inorgchem.4c00679

**Published:** 2024-05-09

**Authors:** Catriona A. Crawford, Craig I. Hiley, Cameron A. M. Scott, Clemens Ritter, Martin R. Lees, Nicholas C. Bristowe, Richard I. Walton, Mark S. Senn

**Affiliations:** † Department of Chemistry, University of Warwick, Gibbet Hill Road, Coventry CV4 7AL, U.K.; ‡ Centre for Materials Physics, Durham University, South Road, Durham DH1 3LE, U.K.; § Institut Laue-Langevin, 71 avenue des Matyrs, CS 20156, 38042 Grenoble cedex 9, France.; ¶ Department of Physics, University of Warwick, Gibbet Hill Road, Coventry CV4 7AL, U.K.

## Abstract

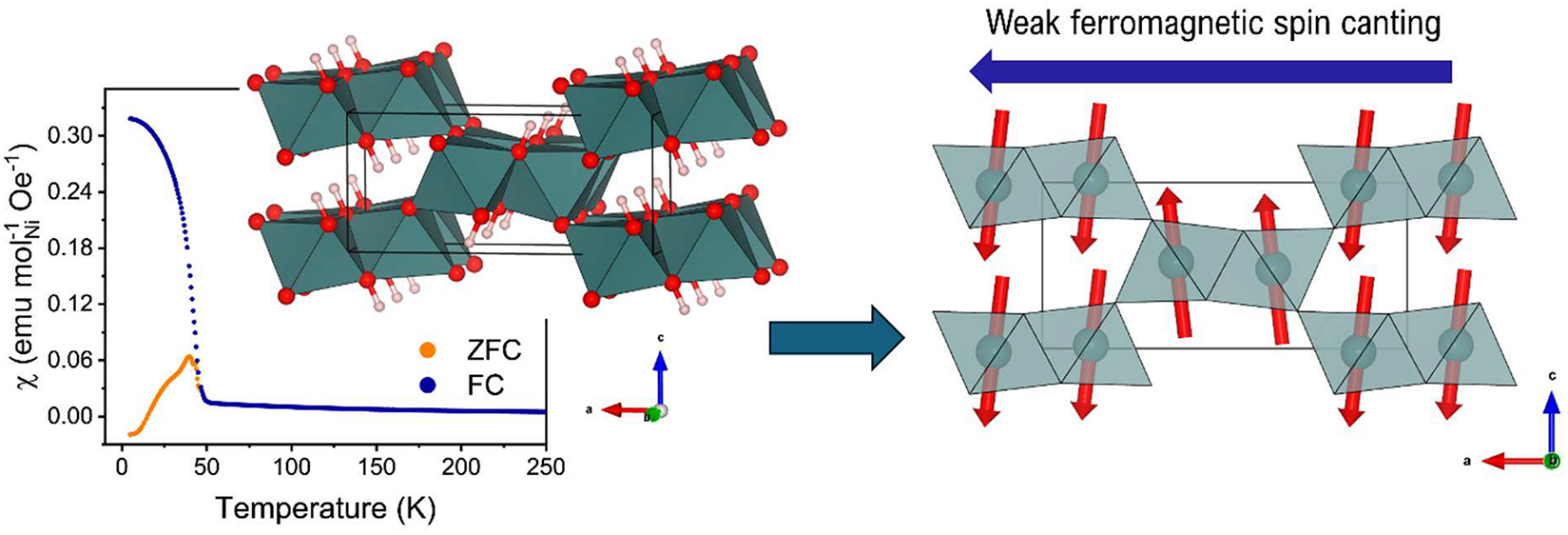

We report a new nickel hydroxyfluoride diaspore Ni(OH)F prepared using hydrothermal synthesis from NiCl_2_·6H_2_O and NaF. Magnetic characterization reveals that, contrary to other reported transition-metal hydroxyfluoride diaspores, Ni(OH)F displays weak ferromagnetism below the magnetic ordering temperature. To understand this difference, neutron diffraction is used to determine the long-range magnetic structure. The magnetic structure is found to be distinct from those reported for other hydroxyfluoride diaspores and shows an antiferromagnetic spin ordering in which ferromagnetic canting is allowed by symmetry. Furthermore, neutron powder diffraction on a deuterated sample, Ni(OD)F, reveals partial anion ordering that is distinctive to what has previously been reported for Co(OH)F and Fe(OH)F. Density functional theory calculations show that OH/F ordering can have a directing influence on the lowest energy magnetic ground state. Our results point toward a subtle interplay between the sign of magnetic exchange interactions, the electronic configuration, and anion disordering.

## Abstract 2

This article discusses the structural origins of weak ferromagnetic behavior in a newly synthesized diaspore, Ni(OH)F. This magnetic behavior differs to that observed in other diaspores which are antiferromagnetic. Through structural characterization on X-ray and neutron diffraction data, we discover a change in the magnetic structure and OH/F anion order between Ni(OH)F and other diaspores. Further computational work highlights an interplay between the anion ordering and magnetic spin arrangement.

## Introduction

Transition metal oxide materials provide a rich compositional playground for tuning magnetic interactions across the 3d series. One way to effectively explore a greater range of transition-metal valence states in solid-state materials is via mixed oxy-fluorides and fluorides that provide opportunity to target lower oxidation states of the transition metal. Additionally, the incorporation of a more electronegative anion (χ_Fluorine_ = 3.98, χ_Oxygen_ = 3.44)^1^ results in changes to the metal–anion bonding characteristics and a possibility of structural variety. Owing to their similar ionic radii, O^2–^ and F^–^ can occupy the same site and are usually disordered throughout a material; however, that does not pose limitations on the variety of different structures found in these mixed anion materials. For example, in the (oxy)fluorides of potassium and titanium, a range of compositions has been reported, including K_2_TiF_6_, K_2_TiOF_4_, K_3_TiOF_5_, and K_7_Ti_4_O_4_F_7_, all of which are synthesized under similar solvothermal synthesis routes.^2^ The structural diversity that comes with mixtures of anions includes the formation of materials that are of lower dimensionality, where connectivity of octahedra may not occur uniformly in three dimensions, one series of which being the oxyfluorotellurates (*M*TeO_3_F, where *M* = Fe, Ga, and Cr)^3^ which form zigzag chains of edge-sharing *MX*_6_ polyhedra that are interconnected through the Te atoms. Ferroelectric properties have also been observed in Pb_5_W_3_O_9_F_10_, which has chains of W octahedra separated by corner-sharing dimers.^4^

**Figure 1:**
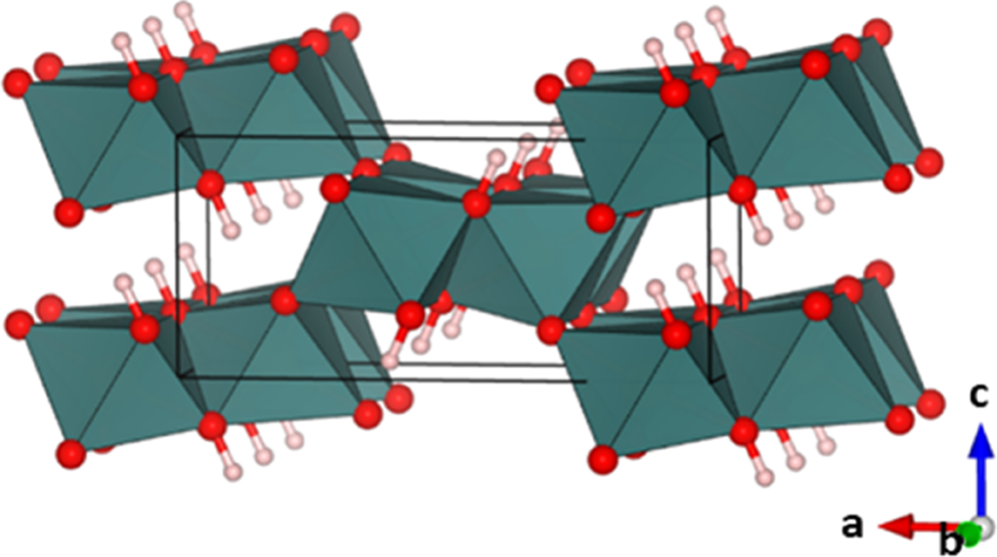
Diaspore structure *M*(OH)*X*, where *M* is located within the octahedra, anions and protons are shown as red and light pink spheres respectively. The structure is drawn to highlight the arrangement of edge-sharing dimer chains along *b*.

The diaspore structure (*M*(OH)*X*, where *M* = Al,^5,6^ Ga,^7^ Mg,^8^ Fe,^9−13^ Co,^14,15^ or Zn^16^ and *X* = O^2–^ or F^–^) consists of edge-sharing *M*X_6_ octahedral dimers that form edge-sharing chains along the shortest axis. These dimer chains are interconnected via corners resulting in channels in which the protons reside (Figure 1). It should be noted that while Figure 1 shows the typically observed full ordering of the (OH) and *X* anions, it is possible for anion disorder to occur meaning that (OH) groups may be present on both the edge-sharing and corner-sharing sites.

When *M* has unpaired electrons, the combination of both 90° and 180° *M*–*X*–*M* bond angles may create competition between the ferromagnetic (FM) and antiferromagnetic (AFM) superexchange interactions, as well as with the direct exchange,^17^ hence providing an opportunity to study the effects of electronic configuration and exchange anisotropy on the magnetic behavior. The oxy-hydroxide diaspores, *M*OOH (*M*^3+^ = Al, Fe, Ga), and hydroxyfluoride diaspores, *M*OHF (*M*^2+^ = Mg, Fe, Co, Zn), all crystallize in the space group *Pnma*,^9,11^ and those that have unpaired spins display collinear AFM behavior.^12^ It is possible to tune the magnetic properties of diaspores by changing the (OH) and F ratio, which has been reported in Co(OH)_2–*x*_F_*x*_, where a deviation away from a 1:1 OH:F ratio comes with a subtle distortion of the Co*X*_6_ octahedra, and a resultant change in the AFM transition temperature.^15^

Here, we report the synthesis of a novel nickel hydroxyfluoride diaspore, Ni(OH)F, that is found to have an AFM ground state but with a weak ferromagnetic (wFM) component that arises from spin canting. This behavior differs from that observed in the other known diaspores. A detailed neutron diffraction study combined with a symmetry analysis of the resulting magnetic structure reveals the origin of the wFM as arising from a change of the sign of the mean exchange interaction within the edge-sharing Ni(OH)_3_F_3_ dimers. We explore the origins of this switching, with respect to previously reported diaspores in terms of both the electronic occupancy of the d-orbitals and anion ordering of (OH) and F anions evident from the neutron diffraction data.

## Experimental Methods

### Synthesis

Using hydrothermal methods, Ni(OH)F was synthesized by mixing NiCl_2_·6H_2_O (0.001 mol) and NaF (0.003 mol) in 10 mL of H_2_O. The resulting solution was sealed in a 20 mL Teflon-lined autoclave and heated to 200 °C for 72 h. After cooling, the solid precipitate was isolated by vacuum filtration and washed with ∼10 mL of H_2_O then ∼10 mL of acetone, and subsequently dried for 3 h at 70 °C. For samples prepared for neutron diffraction, a 0.01:0.03 molar ratio of NiCl_2_·6H_2_O and NaF was dissolved in 100 mL of D_2_O and heated in the same manner in a 200 mL autoclave. Several batches were combined to make a sample large enough for neutron diffraction. Co(OH)F was prepared in an equivalent manner for comparison. ***Caution!*** For hydrothermal synthesis using fluorides where there may be a possibility of producing HF, the remaining liquid after filtration should be mixed in a solution containing a Ca^2+^ source such as Ca(OH)_2_ or calcium gluconate to ensure that any liquid to be disposed of is nonhazardous.

### Characterization

Powder X-ray diffraction (PXRD) was used for each batch of samples synthesized to check phase purity. Data were collected on a Panalytical Empyrean equipped with Cu Kα_1,2_ radiation. Room temperature high-resolution synchrotron powder X-ray diffraction data were collected at Beamline I11 at Diamond Light Source (λ = 0.824970(3) Å). Ultrahigh resolution synchrotron X-ray diffraction data were collected at Beamline ID22 (λ = 0.35433788(8) Å) at the European Synchrotron Radiation Facility (ESRF) at 10, 60, and 295 K to investigate any phase transition at lower temperatures and for use in combined structure refinements with room temperature neutron diffraction data. Temperature control was achieved using a Dynaflow ESRF cryostat.

Powder neutron diffraction (PND) data were collected using a deuterated sample on the high-resolution diffractometer D2B (λ = 1.5934(6) Å) and the high flux diffractometer D20 (λ = 1.54331(2) Å) at the Institut Laue Langevin (ILL). The deuterated sample was loaded into a 9 mm cylindrical vanadium can. High-resolution data were collected for 6 h per measurement on D2B at 10, 60, and 295 K. A variable temperature experiment was carried out on D20, in which the higher neutron flux enables weak reflections to be observed and faster measurements to be carried out. Data were collected between 1.8 and 70 K with a collection time of 10 min and a ramp rate of 0.1 K/min, resulting in a measurement every 1 K, and over the range of 70–300 K, a ramp rate of 0.5 K/min, resulting in a measurement every 5 K. A nondeuterated sample (Ni(OH)F) was additionally measured at 295 K on D2B.

Data were analyzed by the Rietveld method using TOPAS Academic version 7.^18^ A symmetry-based approach was used to investigate structural distortions resulting from magnetic and anion ordering by using files generated using ISODISTORT.^19,20^ Due to the tunable off-stoichiometry of OH and F occupancies reported for Co(OH)_2–*x*_F_*x*_, the deuterium occupancies were not constrained in refinements. For combined refinements of a model on both PXRD (ID22) and PND (D2B) data, the data sets were appropriately weighted so that they contributed equally to the refinement where the individual weightings of each data set were defined by “”, and *x* was a different value for each data set. The individual weightings were adjusted by changing *x* until the goodness of fit (GOF) for each dataset were roughly equal.

Infrared spectroscopy was carried out on a Bruker Alpha FTIR spectrometer over a wavenumber range of 4000 to 400 cm^–1^.

### Magnetic Measurements

Magnetic properties were investigated using a Quantum Design MPMS-5S SQUID magnetometer. ∼20 mg of sample was loaded into a gel capsule, which was then held inside a plastic straw. DC magnetic susceptibility measurements were carried out in the temperature range of 2 to 300 K in a zero-field-cooled (ZFC) and 1 kOe field-cooled (FC) modes. Magnetization as a function of applied field data was collected in the magnetic field range of −50 to 50 kOe at temperatures of 2, 10, 20, 40, 60, and 100 K.

### Computational Details

The computational studies of the electronic, magnetic, and structural properties of various polymorphs of Ni(OH)F were conducted using density functional theory, as implemented in the Vienna Ab-initio Software Package (VASP)^21−24^ using the Perdew–Burke–Ernzerhof exchange correlation functional for solids (PBESol).^25^ Self-consistent field calculations were continued until differences in energy were within a tolerance of 10^–8^ eV. Geometry relaxations were continued until the smallest Hellman–Feynman force was less than 10^–3^ eV/Å.

The presence of OH dimers in the system requires an increased number of plane waves, so a 1000 eV cutoff energy was used in plane wave expansions. A Γ-centered Monkhorst-Pack *k*-grid of dimensions 4 × 7 × 9 was chosen, corresponding to a supercell that is doubled along the *b*-axis of the experimentally determined *Pnma* cell. This was done to allow the consideration of magnetic structures with nonzero *k*-vectors. It also allows for consistent energy comparisons between the *Pnma* structure and *Pmc*2_1_ which already has this larger cell.

In the calculations, projector augmented wave pseudopotentials (PAW PPs) were used with the following electrons treated as valence electrons: Ni – 3p^6^3d^8^4s^2^, O – 2s^2^2p^4^, H – 1s^1^, F – 2s^2^2p^5^. For O, H, and F, the harder pseudopotentials were generated by VASP. This is recommended for materials that contain shorter bonds, such as O–H. Dispersion interaction corrections were not included as we observed that both local pairwise corrections and many-body corrections led to less accurate lattice vectors in the isostructural AlO(OH) diaspore.

Finally, the rotationally invariant formulation^26^ of the onsite Hubbard-*U* parameter was used to incorporate the correlation effects between d electrons.

## Results and Discussion

**Figure 2:**
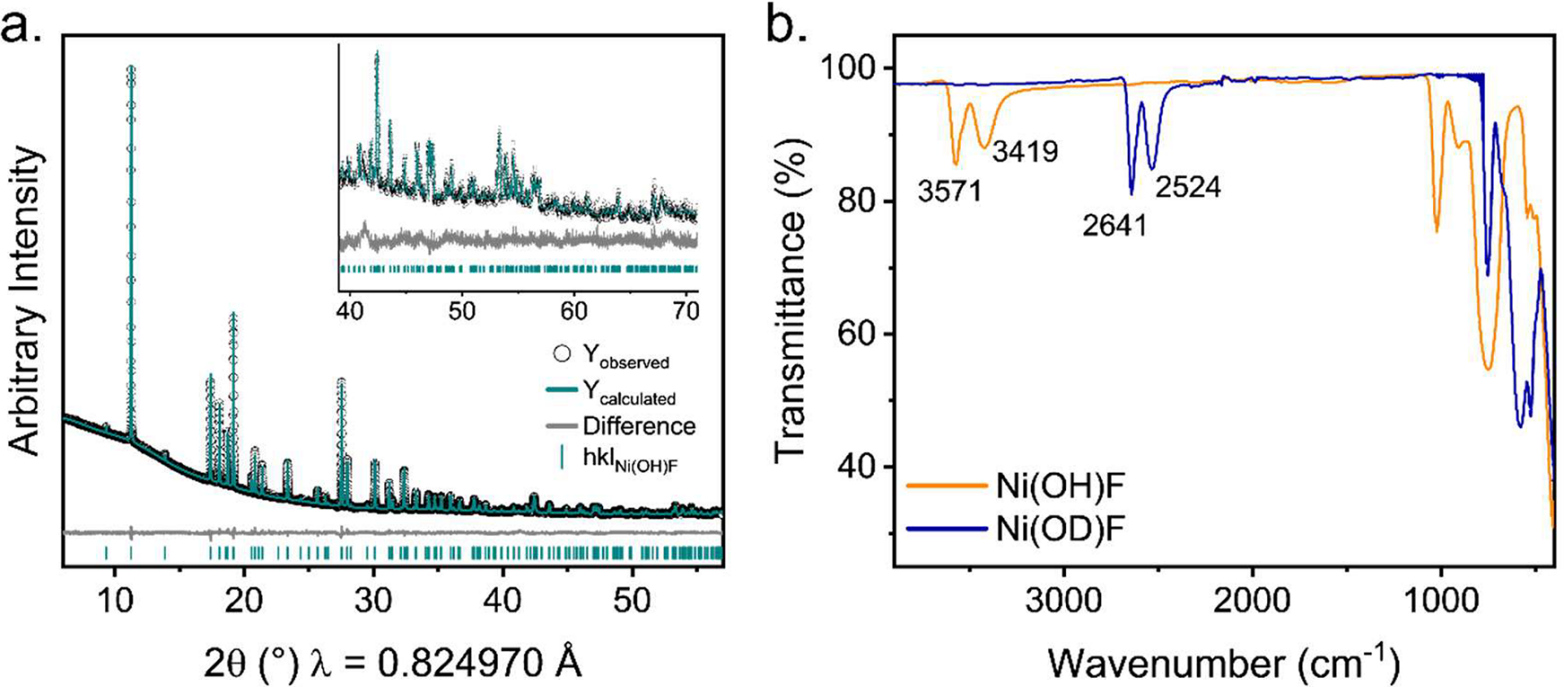
(a) Rietveld refinement on PXRD data of Ni(OH)F collected on Beamline I11 at Diamond Light Source (λ = 0.824970(3) Å). The inset shows the fitting of the high-angle data. (b) An overlay of the IR spectra of samples synthesized in H_2_O (orange) and D_2_O (blue). There is a shift of the characteristic O–H stretches from ∼3500 cm^–1^ to ∼2500 cm^–1^ corresponding to the successful deuteration of the hydroxyl groups.

The synthesized powders were light green in color and were found to crystallize in the centrosymmetric space group, *Pnma* (*a* = 10.1400(3) Å, *b* = 3.06664(7) Å, *c* = 4.61683(1) Å), from a Rietveld refinement achieved against high-resolution PXRD shown in Figure 2a, which is concordant with other reported diaspores. Ni(OH)F could be synthesized with no detectable impurities present at the concentrations described in the experimental methods; however, attempts to increase the amount of product made using increased reagent concentrations resulted in the formation of a NaNiF_3_ impurity. The IR spectra (Figure 2b) were taken for samples synthesized in both D_2_O and H_2_O and sharp bands corresponding to structural OH (OD) could be observed for both samples, indicating the presence of structural hydroxyl groups. Two sharp bands were observed at 3572.06 cm^–1^ and 3424.17 cm^–1^ for the sample synthesized in H_2_O and at 2639.50 cm^–1^ and 2532.19 cm^–1^ for the sample synthesized in D_2_O. The shifting of these peaks to a lower wavenumber in the deuterated sample is confirmative of replacement of OH anions with OD.

**Figure 3:**
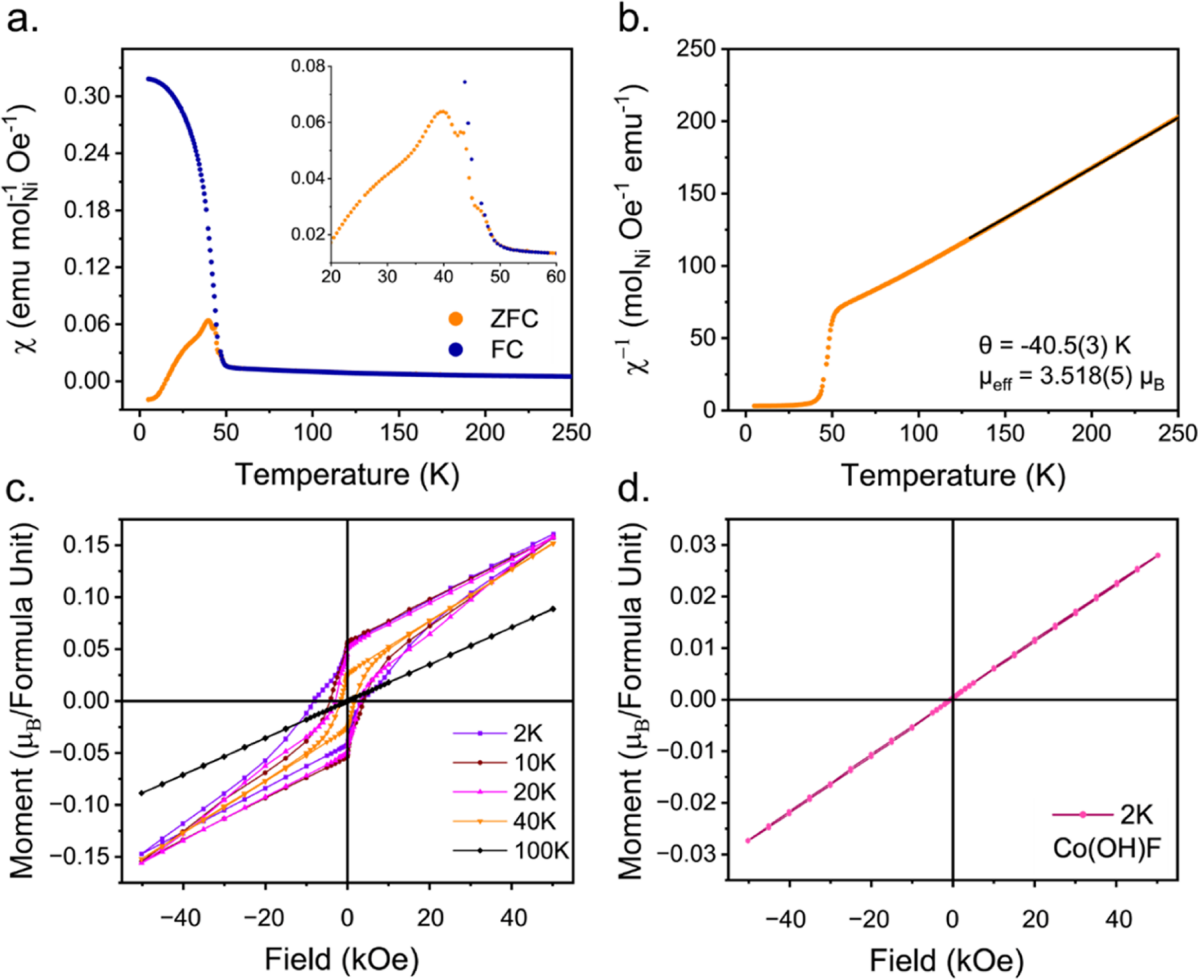
(a) *M*(*T*) of Ni(OH)F showing AFM in the ZFC and FM in the FC measurements. The inset shows a highlighted region where multiple transitions around *T*_N_ can be seen in the ZFC. (b) The inverse susceptibility with a linear fit (black line) to the paramagnetic region. (c) *M*(*H*) loops for Ni(OH)F at 100 K (linear), 40, 20, 10, and 2 K (weak FM hysteresis) with an unsaturated moment. The additional low-temperature FM component is most visible at 2 K. (d) *M*(*H*) loop for Co(OH)F at 2 K showing a linear response, typical of AFM.

The results of the magnetic property measurements for Ni(OH)F are shown in Figure 3. The magnetic susceptibility versus temperature, *M*(*T*), shows Ni(OH)F orders magnetically at a Néel temperature, *T*_N_ = 47(1) K. A cusp in the ZFC data, a clear divergence between the ZFC and FC curves below *T*_N_, and the relatively low magnetic susceptibility are indicative of AFM ordering with a weak FM canting (Figure 3a). The inverse susceptibility (1/χ) in the paramagnetic region (*T* > *T*_N_) follows the Curie–Weiss law (Figure 3b). A linear fit to the high-temperature data yields a θ_CW_ = −40.5(3) K, indicating AFM interactions and a μ_eff_ = 3.518(5) μ_B_/Ni. This effective moment is larger than both the calculated (2.83 μ_B_/Ni) and typically observed (2.9–3.3 μ_B_/Ni) effective magnetic moment for a 3d^8^ ion in an octahedral ligand field ion.^27^ This may be due to an increased magnetocrystalline anisotropy induced by the edge-sharing dimer chains or the presence of short-range FM correlations within the dimers persisting above *T*_N_. A close inspection of the data around *T*_N_ reveals that there are three features in χ(*T*) that are clearly seen in the ZFC curve between 40–50 K (Figure 3a, insert). These may be the result of either magnetic transitions corresponding to OH-rich and F-rich regions which may lead to subtle changes in bond angles and subsequent *M*–*X*–*M* orbital overlap, the ordering of differing components such as a spin alignment along the edge-sharing chains versus alignment between corner-sharing dimers, or AFM ordering followed by a spin canting transition.

Magnetization measurements versus applied field, *M*(*H*), are shown in Figure 3c. Above *T*_N_, a linear response is observed with increasing field, characteristic of paramagnetism at high temperatures. In the measurement at 40 K, below *T*_N_, a weak hysteresis is observed with an *M*_rem_ of ∼0.02 μ_B_ per formula unit. With increasing field strength, the magnetization continues to increase linearly with no evidence of saturation. This again is indicative of AFM with wFM from spin canting. With decreasing temperature, the *M*(*H*) loops adopt a shape that can be described as more wasp-waisted in nature.^28^ Below 10 K, an additional FM component is present at lower applied fields, which increases in size with decreasing temperature. This low temperature feature and the wasp-waisted behavior may be related to a competition between FM and AFM interactions and/or a consequence of magnetic anisotropy.

The magnetization versus applied field can be compared to the compositionally analogous Co(OH)F, which we prepared in the same manner. At 2 K, Co(OH)F shows a linear response to applied field with no hysteresis present (Figure 3d), typical of that observed in AFM materials and in line with previous reports for samples prepared by other methods.^14,15^

**Figure 4:**
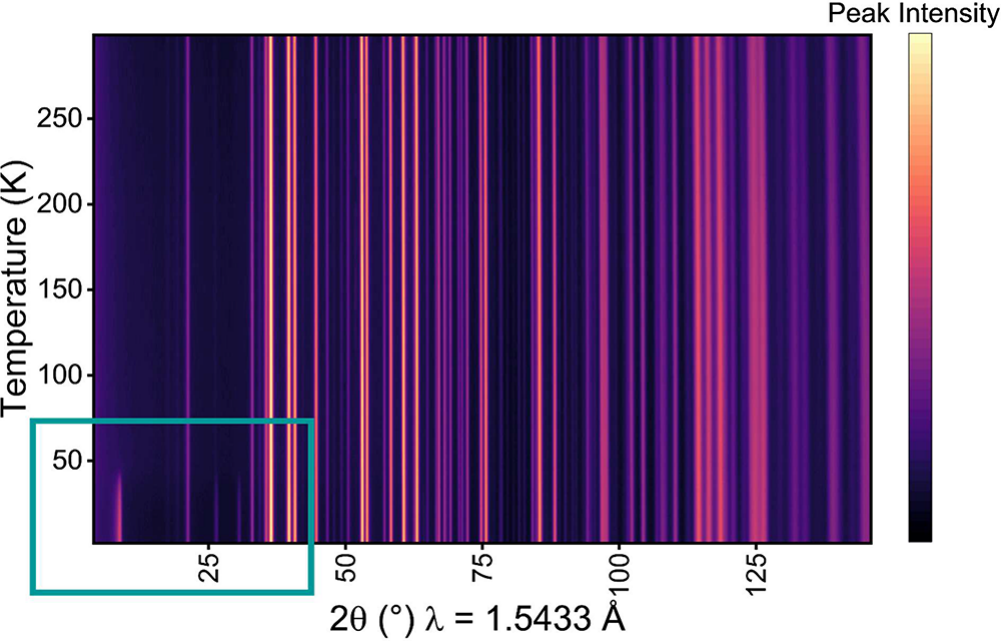
A heatmap of variable temperature PND data collected on D20. The boxed area highlights peaks corresponding to magnetic ordering.

To understand the change in the magnetic behavior from AFM in Co(OH)F, Fe(OH)F, and FeOOH to wFM in Ni(OH)F, neutron diffraction was employed to investigate the magnetic structure. Deuterated Ni(OD)F samples (which shows the same magnetic behavior to Ni(OH)F) were used for all the neutron experiments. Figure 4 shows a heatmap of the variable temperature data collected on D20. Below 50 K, additional reflections corresponding to magnetic ordering are present. These can be indexed to a propagation vector, *k* = [0, 0, 0].

**Figure 5:**
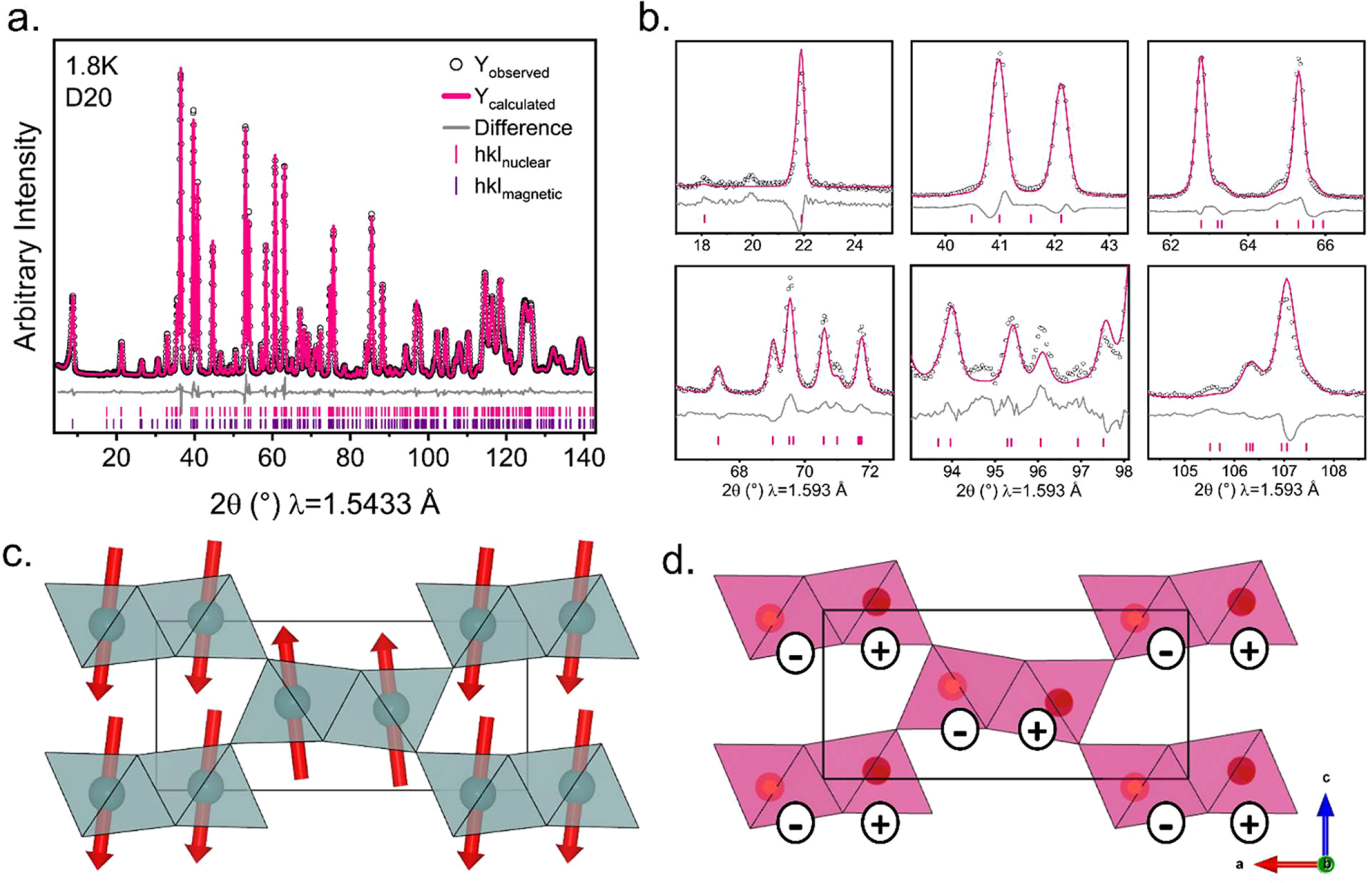
(a) Rietveld refinement of Ni(OD)F in *Pnm′a′* against PND data collected at 1.8 K on D20. (b) Selected peaks from a Rietveld refinement of the model in *Pnma* against PND data collected at 60 K on D2B. (c) The magnetic structure determined experimentally for Ni(OD)F. Spins are coupled FM within dimers and AFM between corner-sharing dimers. (d) The reported magnetic structure (*Pnma′*) for Co(OH)F, Fe(OH)F, and FeO(OH) diaspores. Spins are coupled AFM both within and between dimers. The + and – denote direction of the AFM moments pointing into/out of the plane. Both magnetic structures have FM coupling within the dimer chains along *b*.

Using ISODISTORT, eight irreducible representations (irreps) are identified that the magnetic ordering on the Ni can transform as, leading to eight possible magnetic space groups in *Pnma* in which to model the structure. Data collected at 10 K on the D2B high-resolution powder diffractometer were used for Rietveld refinement. The quality of the fit from Rietveld refinement of the most intense (100) reflection for each subgroup is shown in Supporting Information Figure S1a–h. The magnetic structure was determined as transforming as the irrep mΓ^3+^ in *Pnm′a′* (BNS no. 62.447) and has a magnetic moment of 2.059(9) μ_B_ along *c* directed orthogonal to the dimer chains, where it displays AFM ordering. Along *a* and *b*, the moments are coupled in an FM sense. The refined magnetic moment value against the neutron diffraction data is in good agreement with the expected value of 2 μ_B_ for a d^8^ cation (S = 1). Interestingly, the magnetic space group allows for an FM component along *a*, which provides a likely explanation for wFM observed in our magnetic hysteresis data, corresponding to small spin canting. However due to the expected small magnitude of this (<0.1 μ_B_), the FM components of the moments along *a* were fixed to zero in the refinements. Table 1 shows the information obtained from refinement, and the refinement is shown in Figure 5a.

**Table 1:** Ni(OD)F Structural Parameters from a Magnetic Refinement in *Pnm′a′* at 1.8 K against Neutron Diffraction Data Collected on D20 at the ILL; the Magnitude of Magnetic Moment, *M* (μ_B_/Formula Unit), along *a* Was Fixed to Zero Due to the Small Magnitude Resulting from Spin Canting; Occupancies of (OH) and F Were Fixed to 0.5; A1 and A2 Denote Anion Positions Which Have a Mixed (OH) and F Occupancy

Atom	*x*	*y*	*z*	Occupancy	Site	*B*_iso_(Å^2^)
Magnetic space group: *Pnm′a′*, *R*_wp_ = 2.906%, GOF = 8.407.						
*a* = 10.1206(1) Å, *b* = 3.0592(4) Å, *c* = 4.6068(7) Å, α = β = γ = 90°.						
**Ni**	0.36670(6)	0.25	0.4790(2)	1	4*c*	0.17(2)
*Mx* = 0 *My* = 0 *Mz* = 2.059(9) μ_B_/formula unit						
**A1**	0.5508(1)	0.25	0.2906(3)	O = 0.548	4*c*	0.19(2)
				*F* = 0.452		
**A2**	0.3005(1)	0.75	0.2231(2)	O = 0.429	4*c*	0.21(2)
				*F* = 0.571		
**D1**	0.5696(2)	0.25	0.0928(5)	0.548(5)	4*c*	1.35(7)
**D2**	0.6325(3)	0.25	0.9371(5)	0.429(4)	4*c*	1.03(5)

On close inspection of the data, there are subtle shoulders on various peaks and weak reflections in the neutron diffraction data that are unaccounted for in *Pnma* and could not be attributed to an impurity phase. These are emphasized in PND data collected on the higher resolution D2B instrument, and selected peaks are highlighted in Figure 5b. These peaks are present at all temperatures and are therefore unrelated to the magnetic ordering, and, as we will discuss later, arise due to partial anion ordering.

A visual comparison of the magnetic structure determined for Ni(OH)F and *M*(OH)F (*M* = Co, Fe) are shown in Figures 5c,d respectively. The comparison makes it evident that there is a change in the magnetic easy axis between the two different magnetic structures. However, more interestingly in Ni(OH)F, the moments are coupled ferromagnetically within the octahedral dimers, whereas in Co(OH)F the only FM coupling occurs along the edge-sharing chains and AFM coupling is observed within the dimers. A symmetry analysis of this magnetic structure (mΓ^2–^*Pnma′*) reveals that no FM component is allowed along any of the orthogonal crystal axes, meaning that spin canting does not occur as a natural consequence of this kind of AFM order observed in Co(OH)F and other diaspores, hence explaining the reason for the difference between the magnetic susceptibilities of *M* = Ni and *M* = Co and Fe. In fact, of the eight possible irrep *k* = [0, 0, 0] magnetic structures, four have allowed spin canting, but only two of these have uncompensated FM canting, with the other two having AFM compensated canting. From our analysis it is evident that spin canting is only present in magnetic space groups where the magnetic easy axis, and therefore alignment of magnetic moments is within the *a*/*c*-plane. Any magnetic structure which has the magnetic moments aligned along the *b*-axis (along the dimer chains, as in *M* = Co) cannot have any spin canting, regardless of whether the dimers have AFM or FM interactions. This therefore points toward the single anion anisotropy associated with the transition metal being the dictating factor as to whether wFM is observed or not.

**Figure 6:**
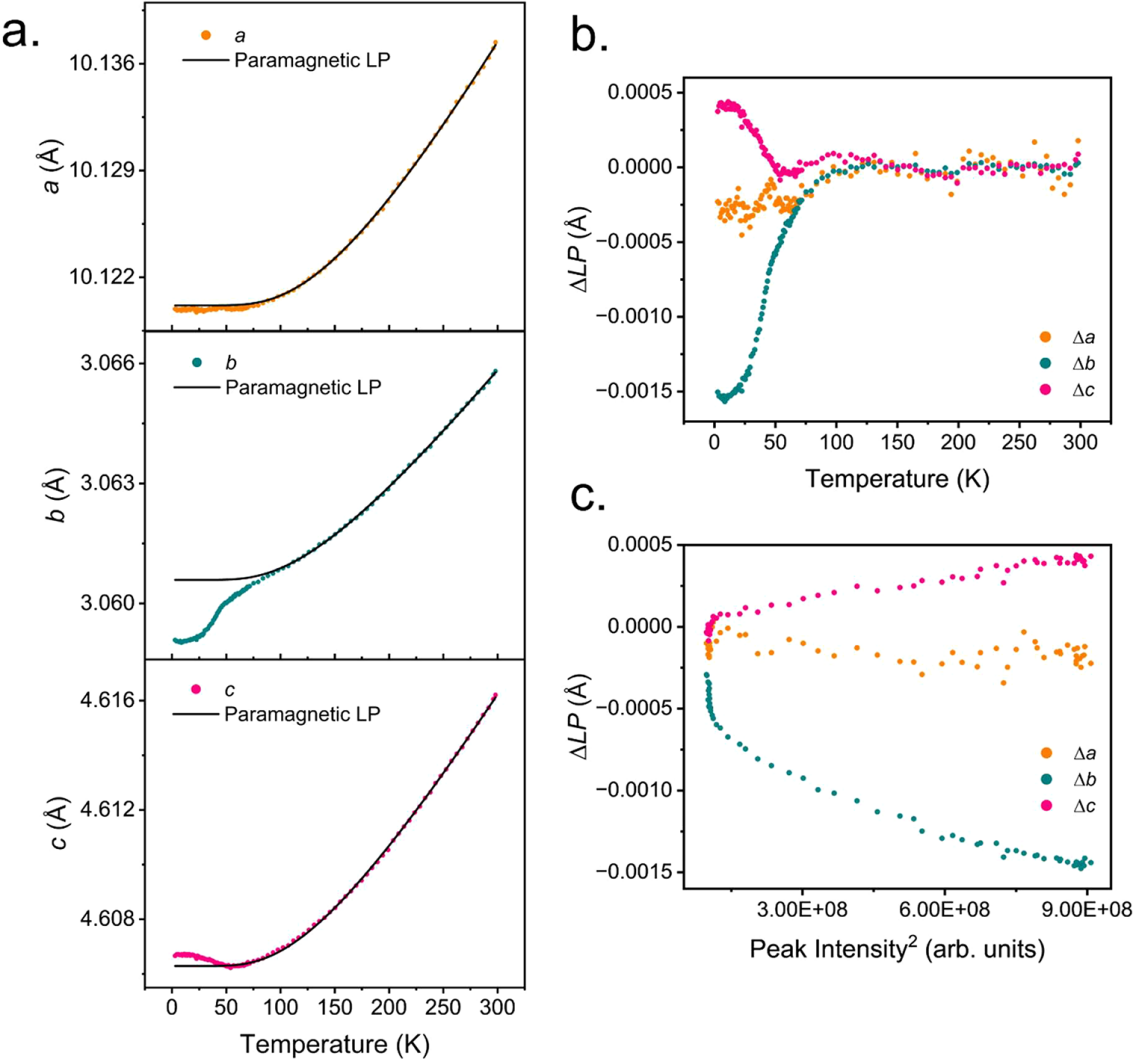
(a) Variations in lattice parameter (LP) with temperature. The paramagnetic region was fitted to an Einstein mode model and extrapolated to 0 K. (b) The excess lattice parameter deviation away from the Einstein mode model. (c) The excess lattice parameter against intensity^2^ of the strongest magnetic peak in PND data.

To study the effects of magnetic ordering on the thermal expansion and contraction of the unit cell, the lattice parameters were extracted from a sequential refinement against the D20 data and plotted against temperature.^29,30^ The paramagnetic region above *T*_N_ was fitted to a single Einstein mode model, represented by  and extrapolated to the lowest temperatures.^31^ There is a clear deviation away from the expected behavior of the lattice parameters in the absence of magnetic ordering, whereby, there is a contraction along the dimer chains (along *b*) and a clear expansion along the *c*-axis as shown in Figure 6a. This deviation (the excess lattice parameters) is shown against temperature, which shows that there is a clear excess negative thermal expansion (NTE) observed along *b* at 50 K (Figure 6b) and is indicative of long-range magnetic exchange interactions that are satisfied below T_N_. Additionally, there is a positive thermal expansion (PTE) along *c*, which may arise to prevent any abrupt any volume change due to the strong NTE along *b*. The magnetic interactions along this direction may also experience some frustration which can also cause PTE. The coupling between the magnetostriction and magnetic order parameter can be investigated by plotting the excess lattice parameter against the intensity of the (100) magnetic reflection (Figure 6c). There is a linear coupling observed; however, along *b* this coupling deviates from linearity below 20 K and can be observed more clearly by the reversal in the magnetostriction in Figure 6b. This decoupling may be related to the low-temperature behavior observed in the *M*(*H*) measurements (Figure 3c).

**Figure 7:**
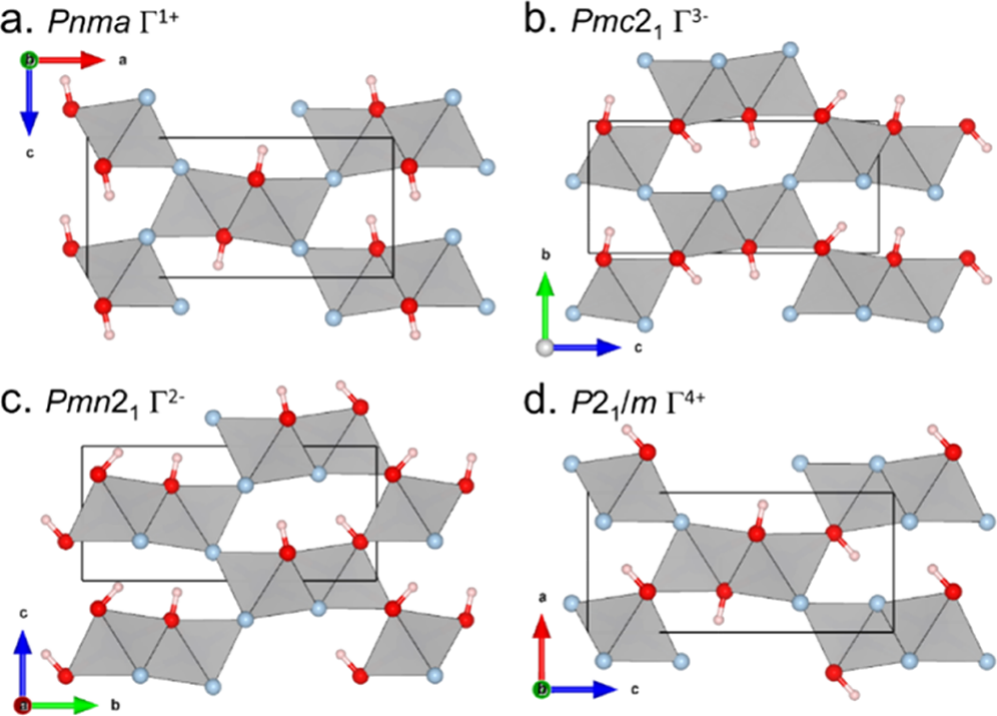
Anion ordering models identified through ISODISTORT: (a) *Pnma*, (b) *Pmc*2_1_, (c) *Pmn*2_1_, and (d) *P*2_1_/*m*. The models in *Pmc*2_1_, *Pmn*2_1_, and *P*2_1_/*m* are displayed with only contributions from their respective gamma points (Γ^3–^, Γ^2–^, and Γ^4+^, respectively); however, they all can additionally contain possible contributions from the Γ^1+^ mode, which affects the relative occupancies between edge-sharing and corner-sharing anion positions. Unit cells have been expanded to highlight the resulting anion ordering. Oxygen atoms are shown in red, protons in pink, and fluorines in light blue. The gray polyhedra represent the Ni*X*_6_ octahedra.

To further understand the appearance of additional reflections in the PND data, symmetry lowering through other possibilities of anion ordering were investigated. Anion ordering was probed through the location and occupancy of the deuterium ion from the neutron diffraction data as the near-identical neutron and X-ray scattering lengths of oxygen and fluorine mean that they cannot be distinguished from each other. Four limiting anion ordering models, which have the propagation vector *k* = [0, 0, 0] consistent with the absence of any superstructure reflections implying cell doubling, were identified using ISODISTORT (shown in Figure 7a–d) and are represented by their space groups: *Pnma*, *Pnm*2_1_, *Pmc*2_1_, and *P*2_1_/*m*.

**Figure 8:**
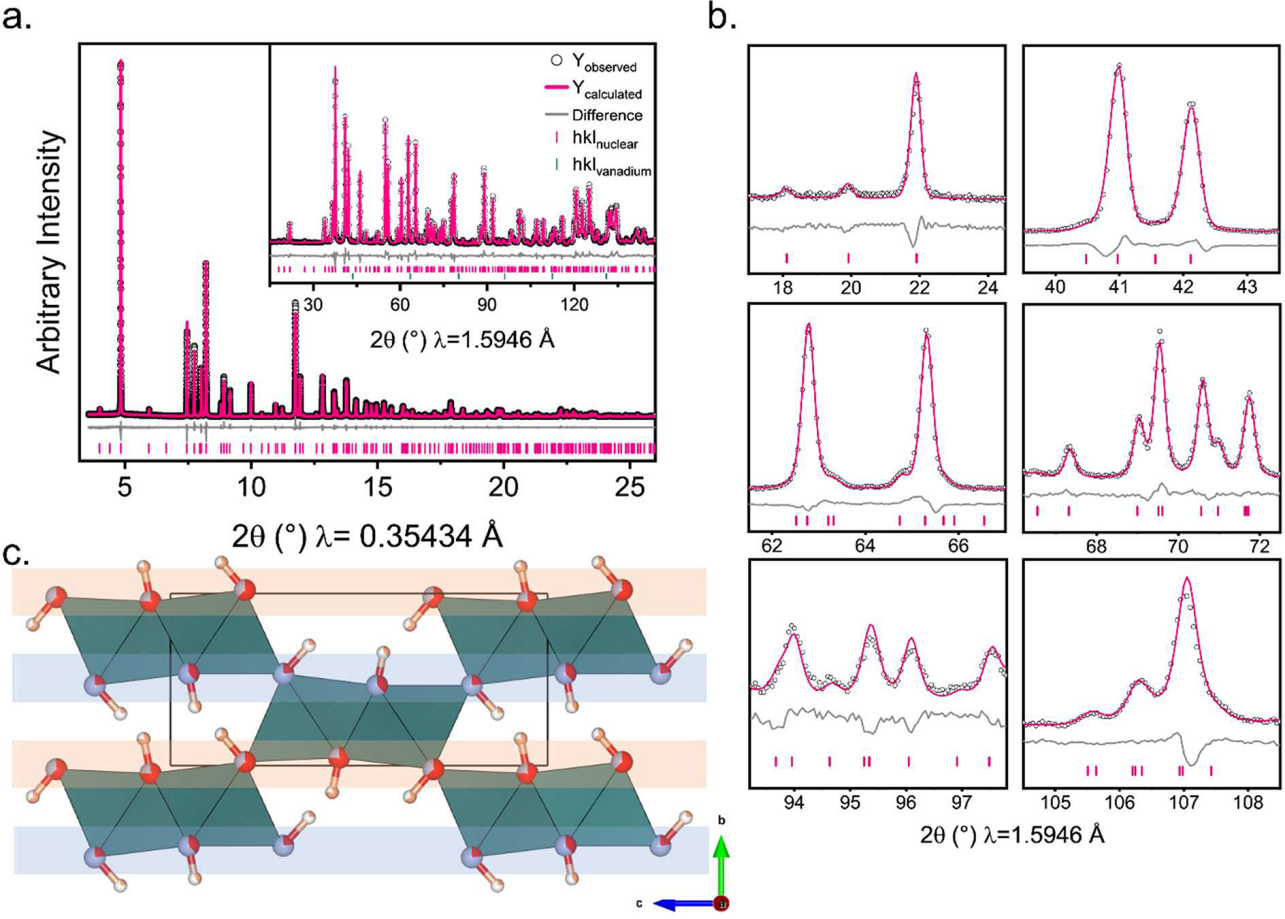
(a) Rietveld refinement of Ni(OD)F in *Pmc*2_1_ at 60 K with a partial ordering of (OD) and F from a combined refinement against PXRD data collected at ID22 (main panel) and PND data collected on D2B (inset). (b) Selected peaks from a Rietveld refinement of the model in *Pmc*2_1_ against PND data collected at 60 K on D2B. (c) Resulting structure with ‘F-rich’ (blue) and ‘OH-rich’ (orange) layers highlighted. Superstructure peaks used to determine the symmetry as *Pmc*2_1_ could not be fitted to any impurity phase.

To investigate the suitability of each model, symmetry mode refinements were performed, and by varying the mode amplitude that corresponded to the deuterium occupancy at fixed values, it is possible to observe trends in the goodness of fit for each model (Supporting Information Figure S2). While the best fits for all four models occur at nonzero mode amplitudes (implying that there is some degree of anion ordering), only one model, *Pmc*2_1_, has allowed reflections that fit the additional peaks not fitted in *Pnma.* A combined neutron and X-ray refinement on data collected at 60 K on D2B and ID22, respectively, is shown in Figure 8a, with additional peaks highlighted in Figure 8b. The 60 K data were used to avoid contributions to peak intensity from magnetic scattering below the magnetic transition temperature in the PND data. Table 2 shows the refinement details and number of refined parameters. The best refinement does not correspond to a ‘fully ordered’ anion model but has a degree of disorder over the anion positions, with fluorine-rich sites and OH-rich sites with an overall composition of Ni(OH)_0.98_F_1.02_. These F/OH-rich sites layer themselves in alternating fashions as shown in Figure 8c. It should be noted that upon reducing the structural symmetry, the combination of two primary order parameters, mΓ^3+^(magnetic ordering) and Γ^3–^(anion ordering in *Pmc*2_1_) induces a secondary magnetic order parameter, mΓ^1–^, that we find no evidence of in our data. This additional order parameter would likely lead to a modulation of the magnitude of the Ni moment due to differing OH/F environments on the two metal sites within the dimers. However, these additional modulations are constrained to be AFM in nature, and so cannot account for the observed wFM. Additional refinements were performed on the deuterated sample at room temperature (Table S1) and on the nondeuterated sample, Ni(OH)F, which had similar results that are provided in Supporting Information Figure S3 and Table S2.

**Table 2:** Ni(OD)F Results from a Combined Refinement in *Pmc*2_1_ at 60 K against Neutron and X-ray Diffraction Data (D2B and ID22, Respectively); Atomic Positions and Occupancies Were Refined Using Symmetry Modes Generated by ISODISTORT, with Non-stoichiometry of the Deuterium Occupancy Allowed; O and F Occupancies Were Changed to Reflect the Resulting Occupancy Deuterium Occupancy on the Associated Site

Atom	*x*	*y*	*z*	Occupancy	Site	*B*_iso_(Å^2^)
Space Group: *Pmc*2_1_, *R*_wp_ = 5.843%, GOF = 1.507.						
*a* = 3.0623(2) Å, *b* = 4.6095(4) Å, *c* = 10.1252(1) Å, α = β = γ = 90°.						
**Ni1**	0	0.2272(2)	0.3614(2)	1	2*a*	0.18(2)
**Ni2**	0.5	0.2679(2)	0.6284(2)	1	2*b*	0.11(2)
**A1**	0	0.0471(5)	0.5485(3)	O = 0.76	2*a*	0.11(4)
				F = 0.24		
**A2**	0.5	0.4695(5)	0.4478(3)	O = 0.37	2*b*	0.45(5)
				F = 0.63		
**A3**	0	0.5347(6)	0.6964(3)	O = 0.22	2*a*	0.81(5)
				F = 0.78		
**A4**	0.5	–0.0226(6)	0.2983(3)	O = 0.62	2*b*	0.19(4)
				F = 0.38		
**D1**	0	0.842(1)	0.5713(5)	0.76(3)	2*a*	1.13(5)
**D2**	0.5	0.659(1)	0.4364(5)	0.37(3)	2*b*	0.87(4)
**D3**	0	0.698(2)	0.6331(8)	0.22(3)	2*b*	0.26(7)
**D4**	0.5	0.818(2)	0.3651(8)	0.62(3)	2*b*	0.66(3)

**Figure 9:**
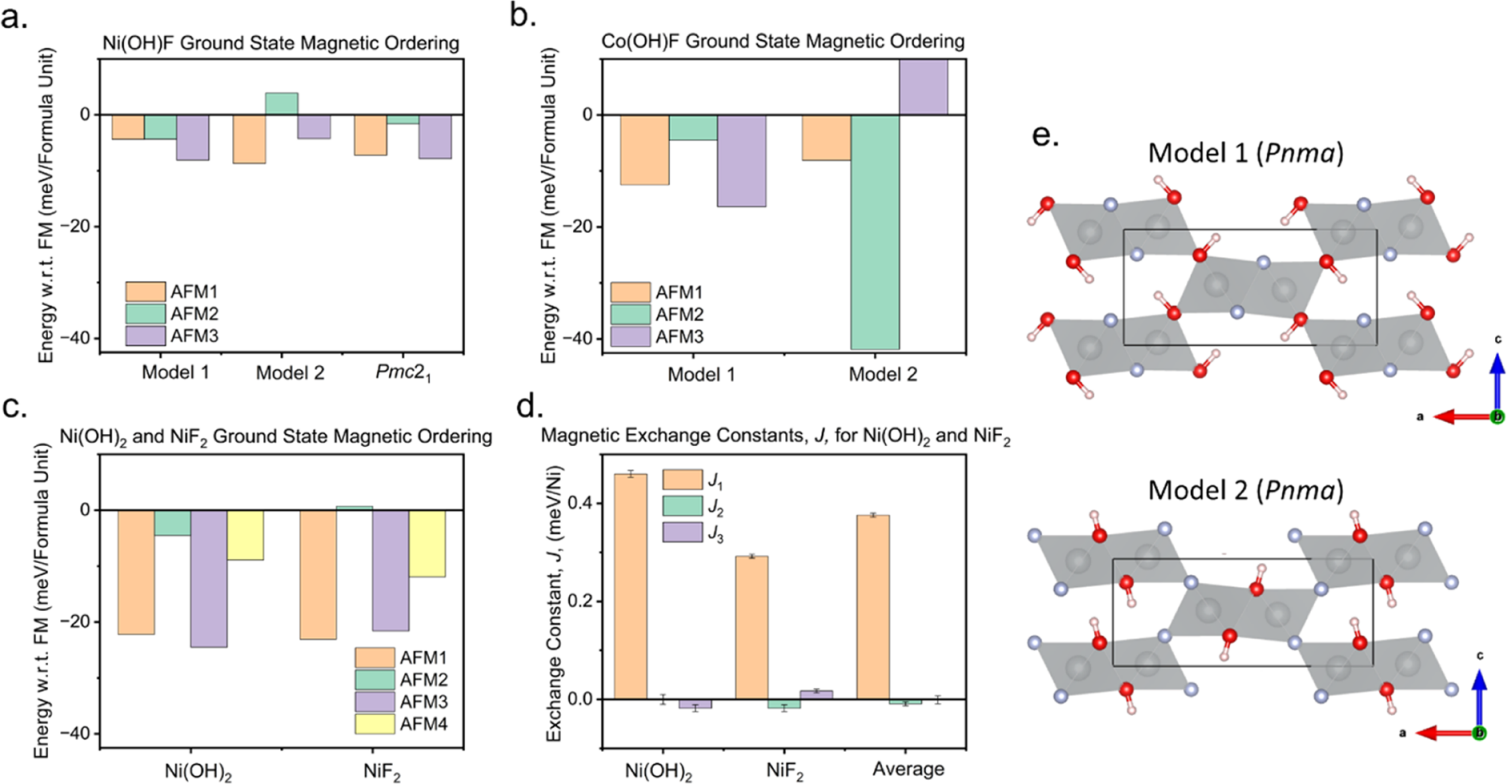
DFT calculations of the diaspores. (a) Ni(OH)F showing a change in the lowest energy ground state between anion ordered **Models 1** and **2**. (b) Co(OH)F showing a change in the lowest energy ground state between anion ordered **Models 1** and **2**. (c) NiF_2_ and Ni(OH)_2_ in the diaspore structure which have different lowest energy ground states. (d) the magnetic exchange constants for Ni(OH)_2_, NiF_2_, and an average. (e) Anion ordered **Models 1** and **2** in *Pnma*. **AFM1** is the experimentally observed magnetic ordering for Ni(OH)F, **AFM2** has AFM within dimers and FM along chains and between dimers, **AFM3** is the experimentally observed magnetic ordering for Co(OH)F, and **AFM4** is AFM along the dimer chains and FM within and between dimers. DFT results are shown with respect to the fully ferromagnetic structure, in which all magnetic interactions are FM.

To obtain a further understanding of the change in magnetic behavior between Ni and the other magnetic diaspores, density functional theory (DFT) calculations were performed. Owing to the difficulty in modeling disorder (for example, in partially occupied H sites), an anion ordered configuration was used. Two anion ordered models in *Pnma* were initially used: **Model 1**, which had OH located at the corner-sharing positions and F located at edge-sharing positions, and **Model 2**, where OH were located along edge-sharing positions, and F atoms were located at corner-sharing anion positions (Figure 9e).

The anion ordering presented in **Model 2** is observed in various other diaspores such as AlOOH, FeOOH, and Mg(OH)F. A third model in *Pmc*2_1_ was used, which is representative of the partial anion ordering observed experimentally by neutron diffraction. To investigate the magnetic ground state, four possible spin arrangements were considered for each of these limiting anion ordering models: **AFM1** which denotes FM within dimers and AFM order between dimers (i.e., the experimentally determined structure for Ni(OH)F), **AFM2** which denotes AFM order within dimers and FM order between corner-sharing dimers, and **AFM3** which has AFM interactions both within and between dimers (i.e., like the reported magnetic structure for FeOOH, Fe(OH)F and Co(OH)F) and FM in which all magnetic order is FM. These are shown in the SI (Supporting Information Figure S4). Figure 9a–b shows the comparison between the energy of the different spin configurations with respect to the energy associated with a fully FM structure for *M*(OH)F. Our results show that the magnetic ground state changes with the anion ordering model used. For Ni(OH)F, the **AFM1** model (the experimentally determined magnetic ordering) is most stable in anion ordered **Model 2**, and for anion ordered **Model 1**, the **AFM3** model is the most stable (Figure 9a); however, the energy differences are small. Additionally, DFT calculations were performed on the fully ordered *Pmc*2_1_ model, which was based on anion ordering transforming as Γ^3–^ corresponding to ‘fully ordered’ OH and F layers. These show that the two lowest energy magnetic structures for Ni(OH)F, the **AFM3** and **AFM1** magnetic orderings, are within error of each other. DFT calculations on Co(OH)F (Figure 9b), which is reported as having disordered anion sites, show that there is a much more distinct change in the magnetic ground state based on the anion ordering model used. In **Model 1**, **AFM3**, the experimentally determined magnetic structure, has the lowest energy; however, for anion ordered **Model 2**, **AFM2** has the lowest energy configuration. Thus, we can speculate that due to the similarities in energy between the possible ground states in Ni(OH)F (compared to Co(OH)F), there is likely a much more complex interplay between anion ordering and magnetism. SI Table S3 reports the same results for Ni(OH)F with different Hubbard-*U* values. Out of the 12 structures considered for Ni(OH)F (comprising four magnetic structures for each of the three anion orderings), the DFT results still show that the thermodynamically stable structure is **Model 2** anion ordering with **AFM3** magnetic ordering, in contradiction to the experimental results (**AFM1**) and suggesting the presence of disorder is a crucial consideration when discussing the magnetic structure. However, when considering the experimentally observed anion ordering, any difference in energy between **AFM1** and **AFM3** is insignificant, suggesting that further subtle perturbation of the exchange interactions has an influence over the observed ground state.

**Figure 10:**
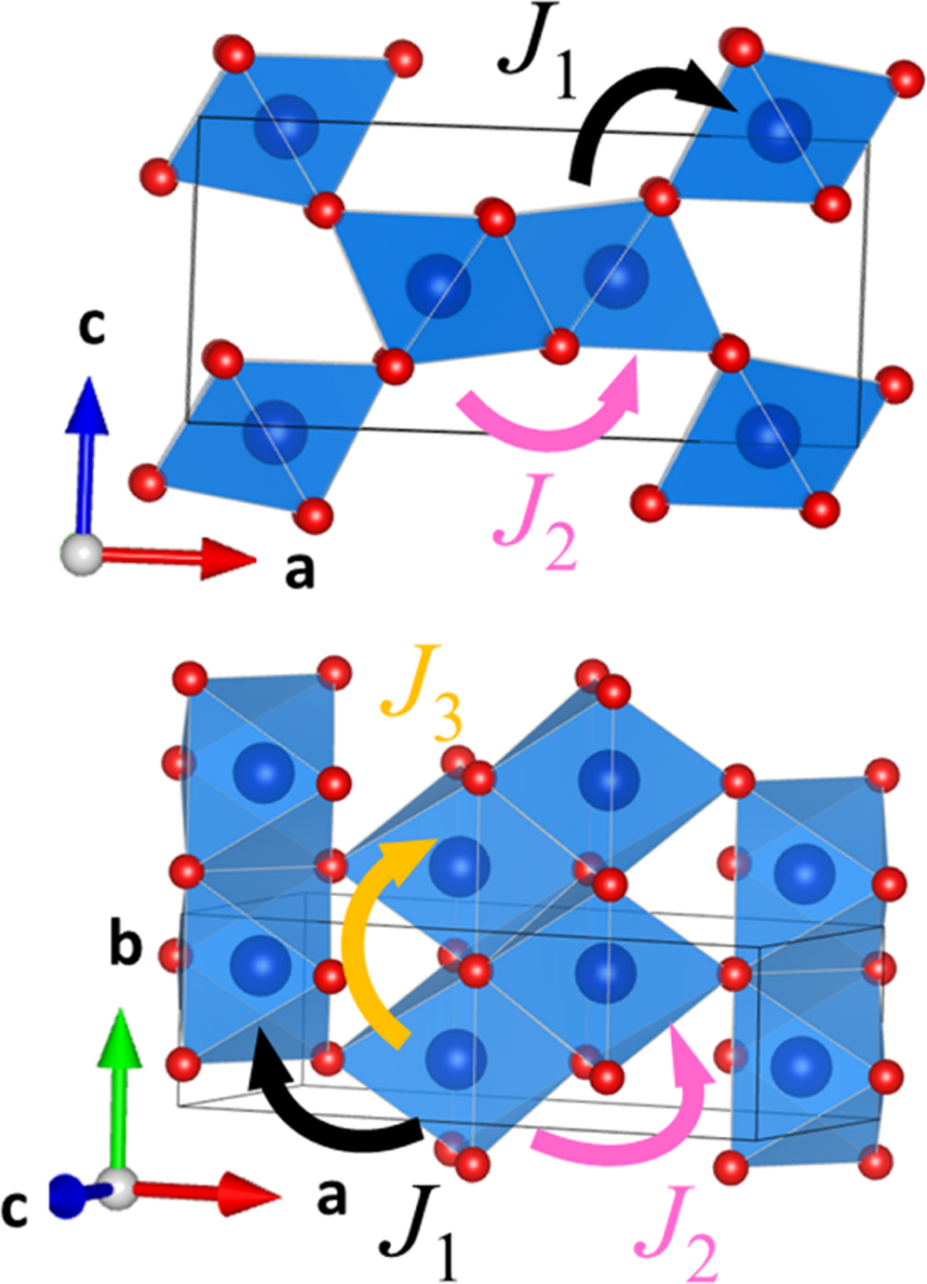
Potential magnetic interactions within the diaspore structure. Black arrows denote a ∼180° AFM superexchange pathway. Orange and pink arrows denote 90° *M*–*X*–*M* bond angles which have the potential for FM exchange. The magnetic coupling constants, *J*, are denoted beside their respective arrows.

In an attempt to further investigate the interplay between anion disorder and long-range magnetic order, we calculated magnetic ground states based on the idealized (hypothetical) NiF_2_ and Ni(OH)_2_ diaspore structures (SI Table S4) and the local exchange interactions: *J*_1_, *J*_2_, and *J*_3_ (Figure 9d, see SI Table S5 for details) for both structures which were averaged to give the expected values for Ni(OH)F. The ground state energies (Figure 9c) clearly suggest there is a selectivity between **AFM1** and **AFM3** dependent on the anion present. The *J*_2,3_ 90° exchange interactions (Figure 10) are very small compared to the AFM *J*_1_, suggesting that we cannot explain these weak interactions by considering their nearest/next-nearest neighbor interactions alone, and the local coordination environment and anions present must also play a role in determining the sign of the magnetic interaction.

Initial interpretation prior to the DFT calculations might have rationalized the change in magnetic structure between Co(OH)F and Ni(OH)F using the Goodenough–Kanamori–Anderson (GKA) rules,^17,32^ whereby we would expect Co^2+^ (d^7^ high spin) to have a strong *M–M* AFM direct exchange within the dimers due to the unpaired electron in the *t*_2g_ orbital, whereas Ni^2+^ (d^8^) only has unpaired *e*_g_ orbitals, meaning that 90 °FM superexchange would dominate the edge-sharing magnetic interactions. However, the DFT results show that the ground-state magnetic structure for both transition metals change based on the anion ordering present, which we find to be distinct in both systems, making a direct comparison difficult. For *M* = Ni, the change in energy between different anion and magnetic ordering modes are very small, making it challenging to disentangle the effects of any partial anion ordering. However, the stronger influence of anion ordering on magnetic ground state of *M* = Co means that the experimentally determined magnetic structure provides a strong indicator as to the likely presence of previously undetected partial or full anion order.

One fact not considered in our DFT calculations, which do not include spin orbit coupling effects, is the strong single anion anisotropy of Co^2+^. If this anisotropy favors moment alignment along a particular crystallographic axis, this will have a strong influence on the symmetry-allowed exchange interactions, providing a further possible explanation for the differing magnetic exchange interactions observed for the different transition metals.

## Conclusions

A new nickel hydroxyfluoride was synthesized using a HF-free synthesis procedure. Ni(OH)F crystallizes in the diaspore structure observed for other hydroxyfluorides such as Fe(OH)F and Co(OH)F, and oxy-hydroxides AlOOH and FeOOH. The magnetic properties of Ni(OH)F differ to that observed in all other magnetic diaspores, where the presence of a weak ferromagnetic component is observed. Neutron diffraction was employed to understand the nature of the magnetic ordering revealed the magnetic structure, *Pnm′a′*, which has a wFM component allowed by symmetry. This differs from the magnetic structure observed in Co(OH)F and FeOOH, *Pnma′* which does not have any spin canting allowed. In addition, various additional reflections were present which corresponded to symmetry lowering to *Pmc*2_1_ due to the partial ordering of anions (OH and F). DFT calculations highlighted an intrinsic interplay between the magnetic ground state and ordering of the OH and F anions. It was found that varying the anion order can change the magnetic interactions within the dimers and that no completely ordered arrangement of anions in our DFT computational simulation could reproduce our experimental findings. In addition, strong antiferromagnetic interactions between dimers were observed, explaining the prevalence of the AFM structure of other reported analogues. The occurrence of the novel **AFM1** magnetic structure observed in Ni(OH)F appears to coincide with the global symmetry breaking through anion ordering. This opens the question of how magnetic order is influenced by the local anion coordination around the magnetic cation, and more generally the interplay between anion disorder and magnetic ordering in mixed-anion materials.

## Supporting Information Available

Further experimental details of computational methods, models and results, and additional information for various magnetic and anion ordered refinements (PDF)

## References

[ref1] Allred A. L. Electronegativity Values from Thermochemical Data. J. Inorg. Nucl. Chem. 1961, 17 (3–4), 215–221. 10.1016/0022-1902(61)80142-5.

[ref2] Sheng J.; Tang K.; Cheng W.; Wang J.; Nie Y.; Yang Q. Controllable Solvothermal Synthesis and Photocatalytic Properties of Complex (Oxy)Fluorides K_2_TiOF_4_, K_3_TiOF_5_, K_7_Ti_4_O_4_F_7_ and K_2_TiF_6_. J. Hazard. Mater. 2009, 171 (1–3), 279–287. 10.1016/j.jhazmat.2009.05.141. 19559527

[ref3] Laval J. P.; Jennene Boukharrata N.; Thomas P. New Oxyfluorotellurates(IV):MTeO_3_F (M = FeIII, GaIII and CrIII). Acta. Crystallogr. C. 2008, 64 (2), i12–i14. 10.1107/S0108270107062683. 18252985

[ref4] Abrahams S. O.; Marsh P.; Ravez J. Ferroelectric Structure and Related Properties of Pb_5_W_3_O_9_F_10_. J. Chem. Phys. 1987, 87 (10), 6012–6020. 10.1063/1.453525.

[ref5] Juan-Farfán R. E. S.; Bayarjargal L.; Winkler B.; Haussühl E.; Avalos-Borja M.; Refson K.; Milman V. Pressure Dependence of the Lattice Dynamics of Diaspore, α-AlO(OH), from Raman Spectroscopy and Density Functional Perturbation Theory. Phys. Chem. Miner. 2011, 38 (9), 693–700. 10.1007/s00269-011-0442-3.

[ref6] Sugiura T.; Arima H.; Nagai T.; Sugiyama K. Structural Variations Accompanied by Thermal Expansion of Diaspore: In-Situ Single-Crystal and Powder X-Ray Diffraction Study. Phys. Chem. Miner. 2018, 45 (10), 1003–1010. 10.1007/s00269-018-0981-y.

[ref7] Li S. J.; Zheng C.; Lobring K. C. Refinement of the Crystal Structure of Gallium Oxide Hydroxide, GaO(OH). Z. Kristallogr. NCS. 2003, 218, 11–12. 10.1524/ncrs.2003.218.1.11.

[ref8] Crichton W. A.; Parise J. B.; Müller H.; Breger J.; Marshall W. G.; Welch M. D. Synthesis and Structure of Magnesium Hydroxide Fluoride, Mg(OH)F: A Topological Intermediate between Brucite- and Rutile-Type Structures. Mineral Mag. 2012, 76 (1), 25–36. 10.1180/minmag.2012.076.1.25.

[ref9] Yang H.; Lu R.; Downs R. T.; Costin G. Goethite, α-FeO(OH), from Single-Crystal Data. Acta. Crystallogr. Sect. E 2006, 62 (12), i250–i252. 10.1107/S1600536806047258.

[ref10] Scheinost A. C.; Stanjek H.; Schulze D. G.; Gasser U.; Sparks D. L. Structural Environment and Oxidation State of Mn in Goethite-Groutite Solid-Solutions. Am. Mineral. 2001, 86 (1–2), 139–146. 10.2138/am-2001-0115.

[ref11] Szytuła A.; Burewicz A.; Dimitrijević Ž.; Kraśnicki S.; Rżany H.; Todorović J.; Wanic A.; Wolski W. Neutron Diffraction Studies of α-FeOOH. Phys. Status Solidi (b) 1968, 26 (2), 429–434. 10.1002/pssb.19680260205.

[ref12] Forsyth J B; Hedley I G; Johnson C E The Magnetic Structure and Hyperfine Field of Goethite (α-FeOOH). J. Phys. C: Solid State Phys. 1968, 1, 179 10.1088/0022-3719/1/1/321.

[ref13] Zepeda-Alarcon E.; Nakotte H.; Gualtieri A. F.; King G.; Page K.; Vogel S. C.; Wang H. W.; Wenk H. R. Magnetic and Nuclear Structure of Goethite (α-FeOOH): A Neutron Diffraction Study. J. Appl. Crystallogr. 2014, 47 (6), 1983–1991. 10.1107/S1600576714022651.

[ref14] Ben Yahia H.; Shikano M.; Tabuchi M.; Kobayashi H.; Avdeev M.; Tan T. T.; Liu S.; Ling C. D. Synthesis and Characterization of the Crystal and Magnetic Structures and Properties of the Hydroxyfluorides Fe(OH)F and Co(OH)F. Inorg. Chem. 2014, 53 (1), 365–374. 10.1021/ic402294g. 24328324

[ref15] Zhang Y.; Xu H.; Liu H.; Seehra M. S.; Wang Z.; Li Y.; Li W. Magnetic Ground State and Tunable Néel Temperature in the Spin 1/2 Linear Chain Antiferromagnet Co(OH)_(2–x)_F_x_. Phys. Status Solidi (b) 2022, 259 (4), 2100438 10.1002/pssb.202100438.

[ref16] Serier H.; Gaudon M.; Demourgues A.; Tressaud A. Structural Features of Zinc Hydroxyfluoride. J. Solid State Chem. 2007, 180 (12), 3485–3492. 10.1016/j.jssc.2007.10.007.

[ref17] Khomskii D. I.; Kugel K. I.; Sboychakov A. O.; Streltsov S. V. Role of Local Geometry in the Spin and Orbital Structure of Transition Metal Compounds. J. Exp. Theor. Phys. 2016, 122 (3), 484–498. 10.1134/S1063776116030079.

[ref18] Coelho A. A. Topas v7: General Profile and Structure Analysis Software for Powder. 2023.

[ref19] Stokes H. T.; Hatch D. M.; Campell B. J. ISODISTORT, ISOTROPY Software Suite. https://iso.byu.edu/iso/isotropy.php.

[ref20] Campbell B. J.; Stokes H. T.; Tanner D. E.; Hatch D. M. ISODISPLACE: A Web-Based Tool for Exploring Structural Distortions. J. Appl. Crystallogr. 2006, 39 (4), 607–614. 10.1107/S0021889806014075.

[ref21] Blöchl P. E. Projector Augmented-Wave Method. Phys. Rev. B 1994, 50 (24), 17953–17979. 10.1103/PhysRevB.50.17953. 9976227

[ref22] Kresse G.; Furthmüller J. Efficiency of Ab-Initio Total Energy Calculations for Metals and Semiconductors Using a Plane-Wave Basis Set. Comput. Mater. Sci. 1996, 6 (1), 15–50. 10.1016/0927-0256(96)00008-0.

[ref23] Kresse G.; Furthmüller J. Efficient Iterative Schemes for *Ab Initio* Total-Energy Calculations Using a Plane-Wave Basis Set. Phys. Rev. B 1996, 54 (16), 11169–11186. 10.1103/PhysRevB.54.11169. 9984901

[ref24] Kresse G.; Joubert D. From Ultrasoft Pseudopotentials to the Projector Augmented-Wave Method. Phys. Rev. B 1999, 59 (3), 1758–1775. 10.1103/PhysRevB.59.1758.

[ref25] Perdew J. P.; Ruzsinszky A.; Csonka G. I.; Vydrov O. A.; Scuseria G. E.; Constantin L. A.; Zhou X.; Burke K. Restoring the Density-Gradient Expansion for Exchange in Solids and Surfaces. Phys. Rev. Lett. 2008, 100 (13), 136406 10.1103/PhysRevLett.100.136406. 18517979

[ref26] Dudarev S. L.; Botton G. A.; Savrasov S. Y.; Humphreys C. J.; Sutton A. P. Electron-Energy-Loss Spectra and the Structural Stability of Nickel Oxide: An LSDA+U Study. Phys. Rev. B 1998, 57 (3), 1505–1509. 10.1103/PhysRevB.57.1505.

[ref27] Mugiraneza S.; Hallas A. M. Tutorial: A Beginners Guide to Interpreting Magnetic Susceptibility Data with the Curie-Weiss Law. Commun. Phys. 2022, 5 (1), 95–95. 10.1038/s42005-022-00853-y.

[ref28] Magno de Lima Alves T.; Amorim B. F.; Morales Torres M. A.; Bezerra C. G.; Nobrega de Medeiros S.; Gastelois P. L.; Fernandez Outon L. E.; Augusto de Almeida Macedo W. Wasp-Waisted Behavior in Magnetic Hysteresis Curves of CoFe_2_O_4_ Nanopowder at a Low Temperature: Experimental Evidence and Theoretical Approach. RSC Adv. 2017, 7 (36), 22187–22196. 10.1039/C6RA28727A.

[ref29] Chatterji T.; Iles G. N.; Ouladdiaf B.; Hansen T. C. Magnetoelastic Effect in MF_2_ (M = Mn, Fe, Ni) Investigated by Neutron Powder Diffraction. J. Phys.: Condens. Matter 2010, 22 (31), 316001 10.1088/0953-8984/22/31/316001. 21399371

[ref30] Chatterji T.; Ouladdiaf B.; Hansen T. C. The Magnetoelastic Effect in CoF_2_ Investigated by Means of Neutron Powder Diffraction. J. Phys.: Condens. Matter 2010, 22 (9), 096001 10.1088/0953-8984/22/9/096001. 21389428

[ref31] Chen W. T.; Ablitt C.; Bristowe N. C.; Mostofi A. A.; Saito T.; Shimakawa Y.; Senn M. S. Negative Thermal Expansion in High Pressure Layered Perovskite Ca_2_GeO_4_. ChemComm. 2019, 55 (20), 2984–2987. 10.1039/C8CC09614G. 30785134

[ref32] Kanamori J. Superexchange Interaction and Symmetry Properties of Electron Orbitals. J. Phys. Chem. Solids. 1959, 10 (2–3), 87–98. 10.1016/0022-3697(59)90061-7.

