# Supplementary material for: The Interplay of Electronic Configuration and Anion Ordering on the Magnetic Behavior of Hydroxyfluoride Diaspores
Source: Inorg Chem. 2024 May 9;63(20):9184–94. doi: 10.1021/acs.inorgchem.4c00679 (PMC11110014; doi:10.1021/acs.inorgchem.4c00679)
Supplement: Supplementary file 1 — ic4c00679_si_001.pdf [file ic4c00679_si_001.pdf]

Supplementary Information

The Interplay of Electronic Configuration and Anion Ordering on the Magnetic Behaviour of Hydroxyfluoride Diaspores – Supplementary Information

Catriona A. Crawford[a], Craig I. Hiley[a], Cameron A. M. Scott[b], Clemens Ritter[c], Martin R. Lees[d], Nicholas C. Bristowe[b], Richard I. Walton[a], Mark S. Senn[a*]*

[a] Department of Chemistry, University of Warwick, Gibbet Hill Road, Coventry, CV4 7AL, U.K.

[b] Centre for Materials Physics, Durham University, South Road, Durham, DH1 3LE, U.K.

[c] Institut Laue-Langevin, 71 avenue des Matyrs, CS 20156, 38042 Grenoble cedex 9, France.

[d] Department of Physics, University of Warwick, Gibbet Hill Road, Coventry, CV4 7AL, U.K.

*Mark Senn (M.Senn@warwick.ac.uk) and *Richard Walton (R.I.Walton@warwick.ac.uk).

Supplementary information S1.

Details on the calculations of magnetic exchange constants, *J*.

The magnetic exchange constants, *J*, were calculated to attempt to rationalise the change in magnetic ground state between $Ni(OH)_2$ and NiF_2 in $Pnma$. This was done with the expectation that such calculations would provide information into how the cation coordination environment, and the subsequent effect on magnetic exchange pathways, are affected as we alter the anion. The magnetic structure of the fully disordered material could then be rationalised with a weighted average of exchange constants. The three relevant nearest-neighbour exchange interactions are displayed in **Figure 10**. We define the magnetic exchange energy with the Heisenberg interaction

$$\Delta E_{ij} = JS_i \cdot S_j, \qquad \text{(eq. 1)}$$

where S_i represents the spin on site i, $J > 0$ denotes an antiferromagnetic interaction and $J < 0$ denotes a ferromagnetic interaction. For Ni^{2+}, $S = 1$ with each unpaired d^8 electron contributing $S = \frac{1}{2}$ to the Ni spin. The exchange constants (**Figure 10**) for the two systems were extracted from the magnetic energies using:

$$J_1 = \frac{E_{FM} - E_{AFM_1}}{32} = \frac{E_{AFM_2} - E_{AFM_3}}{32}, \qquad \text{(eq. 2)}$$

$$J_2 = \frac{E_{FM} - E_{AFM_2}}{16} = \frac{E_{AFM_1} - E_{AFM_3}}{16}, \qquad \text{(eq. 3)}$$

and

$$J_3 = \frac{E_{FM} + E_{AFM_3} - 2E_{AFM_4}}{32} = \frac{E_{AFM_1} + E_{AFM_2} - 2E_{AFM_4}}{32}. \qquad \text{(eq. 4)}$$

Two expressions exist for each exchange constant as we have four unknowns (three exchange constants and an unimportant constant E_0) within a system of five linearly independent simultaneous equations (one equation each mapping the DFT energies of the magnetic structures to the coupling constants). This allows us to calculate an average coupling constant with an associated error which captures DFT numerical convergence effects as well as the possibility that next-nearest neighbour interactions are non-negligible.

The resulting values of the magnetic exchange constants at U = 6 eV are shown in **Figure 9d**, and for a variety of Hubbard-U, are collated in **Table S3**. It is apparent that the magnetic structure in these two materials is dominated by the strong antiferromagnetic J_1 interaction between the dimers - an effect which is robust against variations in the strength of on-site repulsion U and supports the hypothesis that the inter-dimer interaction is controlled by 180° superexchange. The J_2 and J_3 exchange constants are considerably smaller, suggesting that the 90° superexchange pathway within the dimer does not capture the whole story. Additional computational work carried out to investigate the possibility of e_g orbital ordering, did not elucidate any further understanding and is provided in **Figure S4**. Taking an average of the exchange constants between the $Ni(OH)_2$ and NiF_2 materials produces (at U = 6 eV) the following set of exchange constants,

$$J_{1,avg} = 0.376 \pm 0.004\,, \qquad \text{(eq. 5)}$$

$$J_{2,avg} = -0.009 \pm 0.004, \qquad \text{(eq. 6)}$$

$$J_{3,avg} = -0.001 \pm 0.008, \qquad \text{(eq. 7)}$$

which would produce the observed **AFM1** magnetic structure but with extremely weak coupling within the dimer and along the chains.

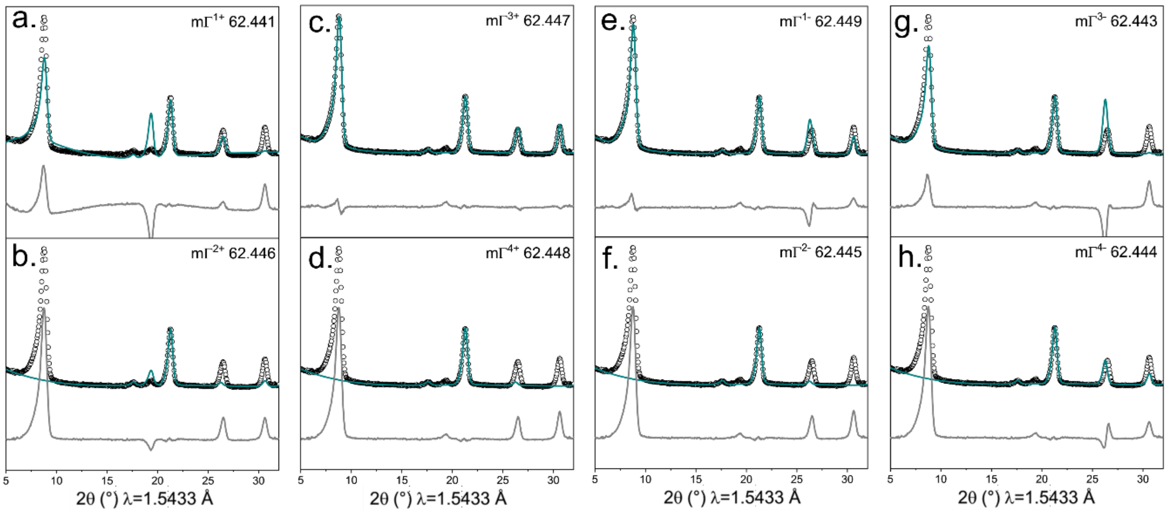

Figure S1. a-h. Rietveld fits of the possible magnetic space groups in *Pnma*. **a, d, f, g** all have magnetic moments aligned along the *b* axis and no spin canting is observed. **b, c, e** and **h** all have magnetic moments that lie in the *a/c* plan, with AFM spin canting in **e** and **h**, and FM spin canting in **b** and **c**, where the only difference between **b/c** is the direction of the large AFM moment along either the *c* or *a* axis respectively. The peak at 19° was later determined to result from symmetry lowering due to anion ordering.

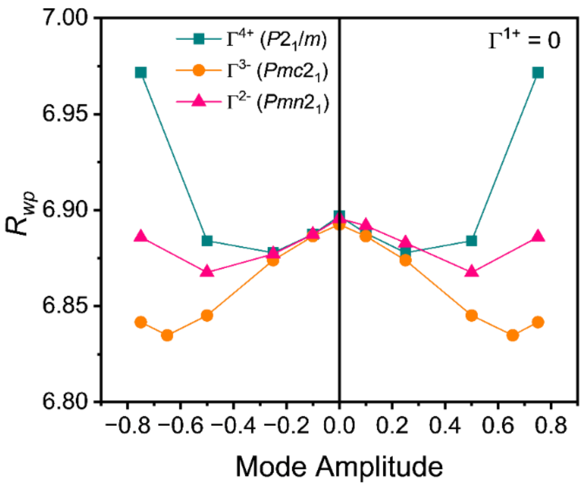

Figure S2. The quality of Rietveld refinement fit to a combined refinement on data on x-ray data collected at Beamline ID22 (ESRF) and neutron diffraction data collected on D2B (ILL) at 60K. The Γ^{1+} mode present in *Pnma* which controls the degree of ordering across two sites

has been set to zero. A mode amplitude of ± 1 indicated a fully ordered system where H occupancies are either 0 or 1. For all possible anion ordering distortions, a non-zero mode amplitude gave the highest quality fit (or lowest R_{wp}).

Atom	*x*	*y*	*z*	Occupancy	Site	B_{iso} (Å^2)
Space Group: *Pmc*2$_1$, R_{wp} = 6.551%, GOF = 1.981. *a* = 3.06776(5) Å, *b* = 4.61933(7) Å, *c* = 10.1411(2) Å, α = β = γ = 90°.						
**Ni1**	0	0.2257(2)	0.3634(2)	1	2*a*	0.44(2)
**Ni2**	0.5	0.2654(2)	0.6306(2)	1	2*b*	0.44(2)
**A1**	0	0.0421(8)	0.5516(3)	O = 0.76<br>F = 0.24	2*a*	0.30(7)
**A2**	0.5	0.4634(8)	0.4518(3)	O = 0.36<br>F = 0.64	2*b*	0.78(8)
**A3**	0	0.5284(9)	0.6996(4)	O = 0.25<br>F = 0.75	2*a*	0.60(8)
**A4**	0.5	-0.0254(9)	0.3005(4)	O = 0.59<br>F = 0.41	2*b*	0.39(7)
**D1**	0	0.848(2)	0.5749(8)	0.76(2)	2*a*	1.43(9)
**D2**	0.5	0.654(2)	0.4460(8)	0.36(3)	2*b*	1.37(3)
**D3**	0	0.687(5)	0.635(2)	0.25(3)	2*b*	1.29(2)
**D4**	0.5	0.813(5)	0.367(2)	0.59(2)	2*b*	1.32(2)

Table S1. Ni(OD)F results from a Rietveld refinement in *Pmc*2$_1$ at 295K against combined neutron diffraction data (D2B) and synchrotron x-ray diffraction data (ID22). Atomic positions and occupancies were refined using symmetry modes generated by ISODISTORT, with non-stoichiometry of the proton occupancy allowed. O and F occupancies were changed to reflect the resulting occupancy deuterium occupancy on the associated site.

Atom	x	y	z	Occupancy	Site	B_{iso} (Å^2)
Space Group: $Pmc2_1$, R_{wp} = 2.617%, GOF = 2.261. a = 3.0672(3) Å, b = 4.6176(6) Å, c = 10.1409(1) Å, α = β = γ = 90°.						
**Ni1**	0	0.2268(8)	0.3802(5)	1	2*a*	0.39(6)
**Ni2**	0.5	0.2631(8)	0.6469(5)	1	2*b*	0.38(5)
**A1**	0	0.035(2)	0.5626(8)	O = 0.76<br>F = 0.24	2*a*	0.55(2)
**A2**	0.5	0.459(2)	0.4628(8)	O = 0.37<br>F = 0.63	2*b*	0.21(2)
**A3**	0	0.525(2)	0.7163(7)	O = 0.22<br>F = 0.78	2*a*	0.90(2)
**A4**	0.5	-0.032(2)	0.3207(7)	O = 0.62<br>F = 0.38	2*b*	0.76(2)
**D1**	0	0.857(3)	0.587(8)	0.76(3)	2*a*	1.08(1)
**D2**	0.5	0.646(3)	0.362(8)	0.37(3)	2*b*	1.44(3)
**D3**	0	0.785(2)	0.564(4)	0.22(3)	2*b*	1.13(3)
**D4**	0.5	0.784(2)	0.393(4)	0.62(3)	2*b*	1.02(1)

Table S2. Ni(OH)F results from a Rietveld refinement in $Pmc2_1$ at 295K against neutron diffraction data (D2B). Atomic positions and occupancies were refined using symmetry modes generated by ISODISTORT, with non-stoichiometry of the proton occupancy allowed. O and F occupancies were changed to reflect the resulting occupancy deuterium occupancy on the associated site.

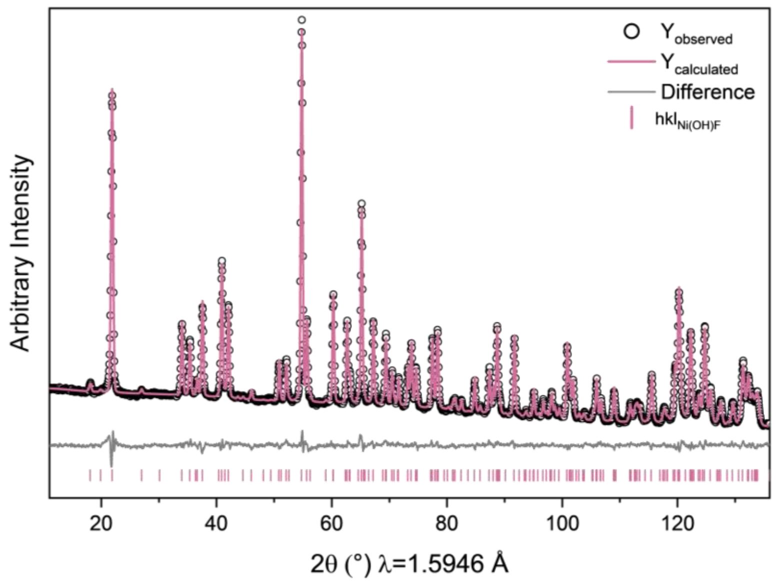

Figure S3. Rietveld refinement of the non-deuterated Ni(OH)F sample in *Pmc*2_1 at 295K with a partial ordering of (OH) and F against PND data collected on D2B.

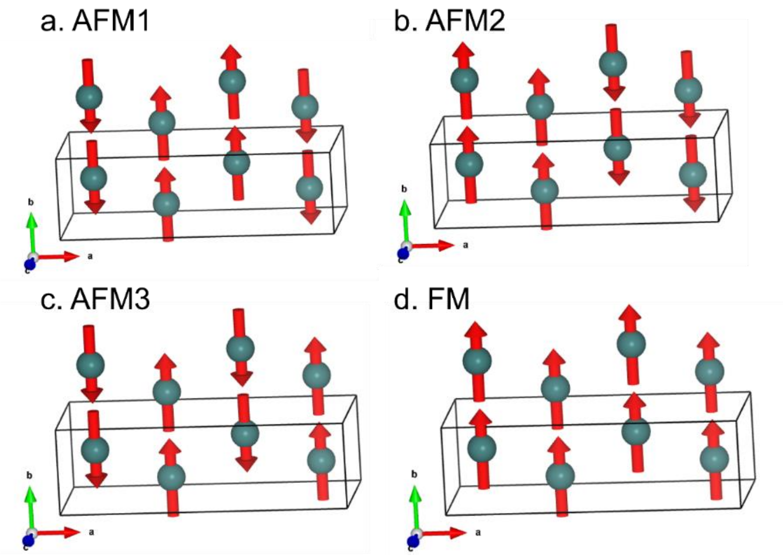

Figure S4. Models used in DFT calculations **a.** AFM1 – the experimentally determined model for Ni(OH)F with FM edge sharing interactions within dimers and AFM corner sharing interactions. **b**. AFM2 with AFM edge sharing interactions and FM corner sharing interactions. **c.** AFM3 – the experimentally determined model for Co(OH)F, Fe(OH)F and FeOOH with AFM along both edge and corner sharing interactions. **d.** All FM interactions. Where results of

the DFT were a positive value, the FM model was more favourable. For this computational work, magnetic moments did not have a direction along a specific unit cell axis and were only defined as ‘spin up’ or ‘spin down’.

Magnetic Structures	*Pnma* Model 1 (eV/f.u)			*Pnma* Model 2 (eV/f.u)			$Pmc2_1$ (eV/f.u)		
	2eV	6eV	8eV	2eV	6eV	8eV	2eV	6eV	8eV
**AFM1**	-20.658	-19.530	-19.103	-20.595	-19.474	-19.048	-20.646	-19.510	-19.077
**AFM2**	-20.657	-19.530	-19.104	-20.568	-19.462	-19.040	-20.634	-19.504	-19.074
**AFM3**	-20.666	-19.534	-19.106	-20.585	-19.470	-19.045	-20.649	-19.510	-19.078
**FM**	-20.647	-19.526	-19.101	-20.576	-19.466	-19.042	-20.627	-19.503	-19.073

Table S3 : DFT energies of the two *Pnma* anion ordering models as well as the alternative $Pmc2_1$ model. Within **Model 1**, **AFM3** is the ground state. Within **Model 2**, **AFM1** is the ground state. Within $Pmc2_1$, **AFM3** is again the ground state but the energies of the two are nearly degenerate. Overall, **Model 1** anion ordering with **AFM3** magnetic ordering is the ground state.

Magnetic Structures	$Ni(OH)_2$ eV/f.u			NiF_2 eV/f.u		
	U=2eV	U=6eV	U=8eV	U=2eV	U=6eV	U=8eV
**AFM1**	-26.382	-25.250	-24.812	-14.284	-13.301	-12.927
**AFM2**	-26.364	-25.235	-24.804	-14.260	-13.291	-12.921
**AFM3**	-26.384	-25.249	-24.812	-14.282	-13.301	-12.927
**AFM4**	-26.369	-25.242	-24.807	-14.272	-13.296	-12.924
**FM**	-26.360	-25.235	-24.803	-14.260	-13.292	-12.921

Table S4. Energies of magnetic structures for the *Pnma* structure for anion sites fully occupied by either F or OH. The variation of these energies with U are also included. For all U, the **AFM1** structure is the ground state for F system and **AFM3** is the ground state for OH.

**Exchange Constant**	**$Ni(OH)_2$ meV/Ni**			**NiF_2 meV/Ni**		
	**U=2eV**	**U=6eV**	**U=8eV**	**U=2eV**	**U=6eV**	**U=8eV**
J_1	0.66(2)	0.460(7)	0.265(4)	0.709(8)	0.292(4)	0.189(2)
J_2	0.21(5)	0.00(1)	0.00(1)	-0.07(2)	-0.018(7)	-0.006(5)
J_3	-0.25(3)	-0.018(7)	-0.027(4)	0.057(8)	0.017(4)	0.010(2)

Table S5 : Magnetic exchange constants and their associated errors for *Pnma* structure for anion sites fully occupied by either F or OH. The variation of these constants with U are also included.

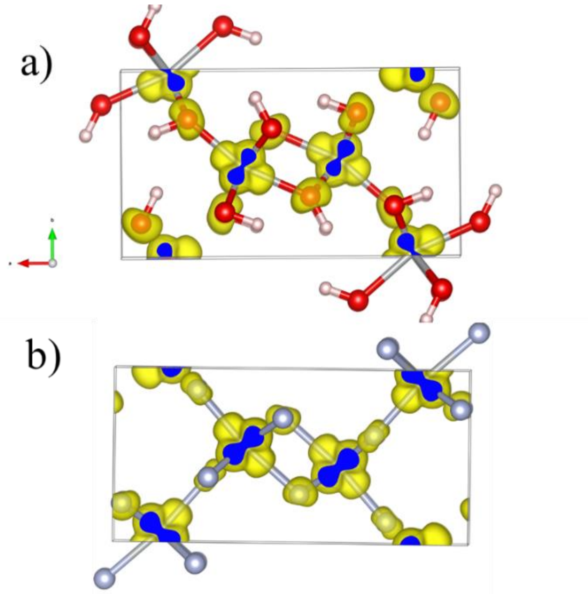

Figure S5. Further calculations investigating the occurrence of e_g orbital ordering. We investigated this by plotting the difference between the up and down spin density. Plotting the difference removes the fully occupied t_{2g} orbitals which do not contribute to the net spin and instead only the e_g orbitals are visualised for **a.** $Ni(OH)_2$ and **b.** NiF_2. Unfortunately, there is no obvious difference between the orbitals in the two structures.
